# Ovarian response to stimulation and suboptimal endometrial development are associated with adverse perinatal outcomes in intracytoplasmic sperm injection cycles

**DOI:** 10.5935/1518-0557.20190001

**Published:** 2019

**Authors:** Edson Borges Jr., Bianca Ferrarini Zanetti, Daniela Paes de Almeida Ferreira Braga, Amanda Souza Setti, Rita Cássia Sávio Figueira, Assumpto Iaconelli Jr.

**Affiliations:** 1 Fertility Medical Group, São Paulo, SP – Brazil; 2 Instituto Sapientiae – Centro de Estudos e Pesquisa em Reprodução Assistida. São Paulo, SP – Brazil

**Keywords:** perinatal outcomes, endometrial thickness, ICSI, COS, SGA

## Abstract

**Objective:**

To study which factors affect perinatal outcomes in intracytoplasmic sperm injection (ICSI) cycles.

**Methods:**

Data was obtained from 402 live births born to 307 patients undergoing ICSI cycles in a private university-affiliated IVF center between Jan/2014 and Dec/2015. The influences of the cycles' characteristics on the number of gestational weeks to livebirth (GW), baby birth weight (BW), and baby birth length (BL) were evaluated by linear regression models, adjusted for maternal age and body mass index, number of transferred embryos, number of gestational sacs, and number of born infants. In a subsequent analysis, GW, BW and baby sex were utilized for cycle classification into the groups Appropriate for gestational age (AGA n=256) and Small for gestational age (SGA n=146), which were compared by general linear models adjusted for the same confounder variables.

**Results:**

The number of follicles (β=-0.069 *p*=0.018) and retrieved oocytes (β=-0.087 *p*=0.049) were negatively correlated with BL. The endometrial thickness was positively correlated with GW (β=0.198 *p*=0.003) and BW (β=28.351 *p*=0.044). When each baby was classified into AGA and SGA groups, it was observed that SGA babies were derived from cycles with higher estradiol levels at hCG day (SGA: 3897.01±550.35 *vs.* AGA: 2324.78±101.86 *p*=0.006) and higher number of retrieved oocytes (SGA: 16.70±1.78 *vs.* AGA: 12.92±0.42 *p*=0.042). The endometrial thickness was significantly lower in the SGA group (SGA: 10.2±0.23 *vs.* AGA: 11.68±0.17 *vs. p*=0.029).

**Conclusion:**

Higher ovarian response to stimulation and suboptimal endometrial development are associated with adverse perinatal outcomes in ICSI cycles.

## INTRODUCTION

Despite assisted reproduction techniques (ART) have advanced significantly since the first in vitro fertilization baby was born, many issues related to the health of ART babies have been raised. Reports comparing babies born from natural pregnancies and those born from assisted reproduction showed correlations between ART pregnancies and worse perinatal outcomes, for instance, preterm birth, low birth weight, small for gestational age, and perinatal mortality (Fauser *et al*., 2014; Helmerhorst *et al*., 2004; Ombelet *et al*., 2016; Pandey *et al.*, 2012; Sunkara *et al*., 2015).

Undoubtedly, intrinsic parental characteristics must influence baby birth and health; however, they are insufficient to explain the differences between babies born from natural or assisted pregnancies, considering that, comparing sons born to the same mother, the ART singleton baby tend to have more perinatal complications than non-ART sibling (Hayashi *et al*., 2012; Henningsen *et al*., 2011; Kapiteijn *et al*., 2006; Pandey *et al*., 2012; Pinborg *et al*., 2013). Moreover, the majority of ART perinatal complications have been commonly attributed to the higher rate of multiple births; nevertheless, singleton pregnancies from assisted reproduction also had significantly worse perinatal outcomes than natural singleton ones (Grady *et al*., 2012; Helmerhorst *et al*., 2004; Pandey *et al*., 2012; Qin *et al*., 2016; Sullivan-Pyke *et al*., 2017). It is plausible that ART perinatal outcomes depend on a complex combination of parental particularities and aspects of the treatment itself (Henningsen *et al*., 2011; Nelson & Lawlor, 2011; Olivennes *et al*., 2002; Palomba *et al*., 2016; Pandey *et al*., 2012; Pinborg *et al*., 2013).

Controlled ovarian stimulation (COS) protocols are pointed as potential influencers of perinatal outcomes (Hayashi *et al*., 2012; Kapiteijn *et al*., 2006). In fact, some studies reported that there was increased number of preterm births and low birth weight babies born to hyper-responder mothers (Kalra *et al*., 2011; Sunkara *et al*., 2015), and that adverse perinatal outcomes may be associated with suboptimal endometrial development due to COS (Chung *et al*., 2006). However, the direct correlation between COS and perinatal outcomes is still characterized by contradictions: when the effects of COS were adjusted for biological and social confounders, such as mother's weight and height, duration of infertility, ethnicity, and level of education, it no longer influenced birth weight and other neonatal characteristics (Griesinger *et al*., 2008; Pelinck *et al*., 2010; Sunkara *et al*., 2016).

In addition to the number of retrieved oocytes, the oocyte quality might have a role in baby development (Balaban & Urman, 2006; Hattori *et al*., 2014; Mateizel *et al*., 2013; Rienzi *et al*., 2011; Shaw-Jackson *et al*., 2014). Embryo quality and embryo transference stage can also influence perinatal outcomes, although direct correlations are not evident (Glujovsky *et al*., 2016; Dar *et al*., 2013; Fernando *et al*., 2012; Källén *et al*., 2010; Kalra *et al*., 2012; Martin *et al*., 2012; Nakagawa *et al*., 2016; Oron *et al*., 2014; Schwärzler *et al*., 2004).

The determination of which aspects of ART pose greater risks of perinatal complications and how these risks can be minimized is extremely important for healthy baby delivery. Therefore, the goal for the present study was to determine which cycle characteristics, for instance, ovarian stimulation, laboratorial, and clinical outcomes, could be correlated with the perinatal outcomes number of gestational weeks to live birth (GW), baby weight at birth (BW), and baby length at birth (BL).

## MATERIAL AND METHODS

### Study design

This cohort study included data obtained from 402 babies born to 307 patients undergoing their first controlled ovarian stimulation (COS) followed by intracytoplasmic sperm injection (ICSI) cycles and fresh transfer of embryos on days three or five of development. Cycles were performed in a brazilian private university-affiliated IVF center between January/2014 and December/2015. Couples undergoing ICSI with vitrified/thawed or donated oocytes, surgical sperm retrieval, vitrified/thawed embryo transfer, donated embryos, or preimplantation genetic diagnosis/screening were excluded from the analysis.

The effects of (i) the total FSH dose administered, (ii) the estradiol peak at hCG day, (iii) the number of follicles, (iv) the number of retrieved oocytes, (v) the number of mature oocytes, (vi) the fertilization rate, (vii) the number of embryos obtained, (viii) the high-quality embryos rate at day two and three, (ix) the blastocyst rate, (x) the transference stage (cleavage or blastocyst), (xi) the endometrial thickness, and (xii) the implantation rate (gestational sacs/ embryos transferred) on the number of GW, BW, and BL were evaluated.

In a subsequent analysis, cycles were subdivided according to the American Academy of Pediatrics Intrauterine Growth Curves (Olsen *et al*., 2010). Combined GW, BW and baby sex were used for the classification of Appropriate for gestational age (AGA), if the baby weight was between 10-90^th^ percentile of the curve (n=256), or Small for gestational age (SGA), if the weight was bellow the 10^th^ percentile (n=146).

Written informed consent, in which patients agreed to share the outcomes of their cycles for research purposes, was obtained, and the local institutional review board approved the study (protocol 410/2012).

### Controlled ovarian stimulation 

Controlled ovarian stimulation was achieved using a daily dose of recombinant FSH (r-FSH, Gonal-F^®^, Merck KGaA, Darmstadt, Germany). Pituitary blockage was performed using a GnRH antagonist (GnRHa, Cetrotide^®^; Merck KGaA, Darmstadt, Germany). Ovulation was triggered with recombinant human chorionic gonadotrophin (hCG, Ovidrel^™^, Merck KGaA, Geneva, Switzerland). Oocyte retrieval was performed 35 hours later through transvaginal ultrasound ovum pick-up.

### Preparation of oocytes

Retrieved oocytes were maintained in culture medium (Global^®^ for Fertilization, LifeGlobal, Connecticut, USA) supplemented with 10% protein supplement (LGPS, LifeGlobal, Connecticut, USA) and covered with paraffin oil (Paraffin Oil P.G., LifeGlobal, Connecticut, USA) for two to three hours before the removal of the cumulus cells. The surrounding cumulus cells were removed after exposure to a HEPES-buffered medium containing hyaluronidase (80IU/mL, LifeGlobal, Connecticut, USA). The remaining cumulus cells were mechanically removed by gently pipetting them with a hand-drawn Pasteur pipette (Humagen Fertility Diagnostics, Charlottesville, USA). Oocyte morphology was assessed using an inverted Nikon Diaphot microscope (Eclipse TE 300; Nikon, Tokyo, Japan) with a Hoffmann modulation contrast system under 400x magnification, just before sperm injection (5 hours after retrieval). Oocytes that had released the first polar body were considered mature and were used for ICSI.

### Intracytoplasmic sperm injection

Intracytoplasmic sperm injection was performed in a micro-injection dish prepared with 4-µL droplets of buffered medium (Global^®^ w/HEPES, LifeGlobal, Connecticut, USA) and covered with paraffin oil on the heated stage of an inverted microscope (37.0±0.5ºC). Sperm selection was performed at 400x magnification. Approximately 16 hours after ICSI, the presence of two pronuclei and the extrusion of the second polar body confirmed fertilization. Embryos were maintained in a 50-µL drop of culture medium (Global^®^, LifeGlobal, Connecticut, USA) supplemented with 10% protein supplement and covered with paraffin oil in a humidified atmosphere under 6% CO_2_ at 37ºC for three to five days.

### Embryo evaluation and transfer

Embryos were morphologically evaluated on days two, three, and five using an inverted Nikon Diaphot microscope with a Hoffmann modulation contrast system under 400X magnification.

High-quality cleavage-stage embryos were defined as those with all of the following characteristics (Alpha Scientists in Reproductive Medicine and ESHRE Special Interest Group of Embryology, 2011): 3−5 cells on day 2 or 8−10 cells on day 3, <15% fragmentation, symmetric blastomeres, the absence of multinucleation, colorless cytoplasm with moderate granulation and no inclusions, the absence of perivitelline space granularity and the absence of zona pellucida (ZP) dimorphisms. The blastocyst rate was defined as the number of embryos that reached blastocyst stage on day five (only the full, expanded, hatching, and hatched blastocyst were considered) by the number of embryos in culture on day three of development.

Embryo transfer was performed on the third or fifth day of development using a soft catheter with transabdominal ultrasound guidance. One to three embryos were transferred per patient, depending on embryo quality and maternal age.

### Clinical follow-up

A pregnancy test (serum β-hCG) was performed 12 days after embryo transfer. All women with a positive test had a transvaginal ultrasound scan after two weeks. Clinical pregnancy was diagnosed when the fetal heartbeat was detected. After childbirth, the GW, BW, BL, baby sex, and presence of malformations at birth were recorded by the patient's gynecologist.

### Statistical analysis

Statistical analysis was performed using SPSS 21 Software (IBM, New York, USA). The effects of response to COS (FSH dose, Estradiol peak at hCG day, number of follicles, number of retrieved oocytes and number of mature oocytes), laboratorial (fertilization rate, number of obtained embryos, high-quality embryo rate at day two and three, blastocyst rate and transference stage), and clinical (endometrial thickness and implantation rate) outcomes on the GW, BW, and BL were evaluated using linear regression models adjusted for maternal age and body mass index (BMI), number of transferred embryos, number of gestational sacs and number of born infants. The results are expressed as β (linear regression coefficient), 95% confidence interval (CI 95%), and *p value*. The α adopted was 5%.

In a subsequent analysis, in which cycles were subdivided AGA and SGA groups and the effects of COS, laboratorial and clinical outcomes were evaluated by general linear models adjusted for maternal age and BMI, number of transferred embryos, number of gestational sacs and number of born infants. The results are expressed as Mean±SD and *p value*. The α adopted was 5%.

## RESULTS

The patient demographics, cycle characteristics, and perinatal outcomes are described in Table 1.

**Table 1:** Descriptive statistics of patients' demographics, cycle characteristics, and perinatal outcomes

	Mean	SD	Range
Paternal age (years)	36.42	4.58	26–49
Maternal age (years)	34.08	3.27	26–40
Maternal BMI (kg/m^2^)	24.46	3.77	17.50 –33.58
Main indication (%)			
Male factor	38.3		
Ovarian factor	12.3		
Tubal factor	15.3		
Endometriosis	14.0		
PCOS	7.8		
Others	12.3		
***COS outcomes***			
Total FSH (IU)	2406.3	525.2	1200 – 3300
Estradiol level at hCG day (pg/mL)	2395.52	1001.69	1046 - 4850
Follicles (n)	15.80	8.00	1 – 38
Retrieved oocytes (n)	12.45	7.21	1–33
MII oocytes (n)	9.19	5.48	1–28
***Laboratorial outcomes***			
Embryos (n)	7.56	4.34	1–20
Fertilization rate (%)	84.53	16.31	13.33 – 100
High-quality embryos rate at day two (%)	81.19	2.13	12.5-100
High-quality embryos rate at day three (%)	65.02	2.43	10.0–100
Blastocyst rate (%)	48.44	2.58	7.0 – 100
Transferred embryos (n)	2.18	0.60	1–3
Transferred embryo stage (%)			
Cleavage-stage	22.6		
Blastocyst	77.4		
***Clinical outcomes***			
Endometrial thickness (mm)	11.07	2.16	7.1 – 17
Gestational sacs (n)	1.53	0.63	1-3
Implantation rate (%)	72.08	26.41	33.33–100
Gestations (%)			
Singleton	73.3		
Twin	24.4		
Triplet	2.3		
***Perinatal outcomes***			
Number of infants (n)	1.48	0.60	1 – 3
Gestational weeks to live birth	36.65	2.44	27–40
Baby birth weight (g)	2709.94	667.75	1040 – 4215
Baby birth length (cm)	46.81	3.36	38–57
Parturition (%)			
Normal	6.5		
Caesarean	93.5		
Baby sex (%)			
Male	54.1		
Female	45.9		
Presence of malformations (%)	0		

PCOS= Polycystic ovary syndrome, COS= Controlled ovarian stimulation, FSH= Follicle-stimulating hormone

The number of follicles (β=-0.069 *p*=0.018) and retrieved oocytes (β=-0.087 *p*=0.049) were negatively correlated with BL (Table 2). The endometrial thickness was positively correlated with the number of GW (β=0.198 *p*=0.003) and BW (β=28.351 *p*=0.044). On average, a 1-mm increase in endometrial thickness could prolong pregnancy by 1.4 days and increase baby weight by 28 g. No correlation between the perinatal outcomes and any other evaluated variable was noted.

**Table 2:** Linear regression predictors for the perinatal outcomes gestational weeks to live birth (GW), baby birth weight (BW) and baby birth length (BL)

	GW			BW			BL		
	β	CI 95%	*p*	β	CI 95%	*p*	β	CI 95%	*p*
Total FSH dose	0.001	0.000;0.001	0.143	0.145	-0.009;0.280	0.156	0.001	0.000;0.002	0.220
Estradiol level at hCG day	0.000	-0.001;0.000	0.261	-0.065	-0.1440.015	0.108	0.000	-0.001; 0.000	0.139
Follicles	-0.005	-0.043; 0.034	0.818	-3.910	-11.207;3.386	0.293	-0.069	-0.127;-0.012	**0.018**
Retrieved oocytes	0.010	-0.024;0.044	0.570	1.060	-5.882;8.002	0.764	-0.087	-0.175;0.000	**0.049**
MII oocytes	-0.004	-0.061;0.053	0.889	-5.649	-16.750;5.451	0.318	-0.029	-0.080;0.022	0.269
Fertilization rate	-0.014	-0.036;0.008	0.209	1.957	-0.334;2.017	0.968	-0.009	-0.042;0.025	0.608
Obtained Embryos	-0.013	-0.089;0.063	0.735	-3.813	-17.800; 10.175	0.592	-0.091	-0.205;0.023	0.117
High-quality embryo rate at day two	0.237	-1.283;1.758	0.759	28.347	-265.482;322.176	0.850	-0.220	-2.610;2.170	0.856
High-quality embryo rate at day three	-0.152	-1.407;1.102	0.811	-1.791	-254.143;250.561	0.989	-0.220	-2.610;2.170	0.856
Blastocyst rate	0.722	-0.596;2.040	0.281	88.885	-185.104;354.87	0.536	-0.973	-3.010;1.064	0.347
Endometrial thickness	0.198	0.069;0.327	**0.003**	28.351	0.770;55.932	**0.044**	0.164	-0.044;0.372	0.121
Implantation rate	0.005	-0.033; 0.042	0.813	0.720	-6.787;8.227	0.850	-0.035	-0.094;0.023	0.238

FSH= Follicle-stimulating hormone, β= Regression coefficient, CI: 95% confidence interval for β. Data was adjusted for maternal age and BMI, number of transferred embryos, number of gestational sacs and number of born infants.

When each baby was classified into AGA and SGA groups, it was observed significantly higher estradiol level at hCG day in SGA babies (3897.01±550.35) compared to AGA group (2324.78±101.86, *p*=0.006) (Table 3). It was also observed a higher number of retrieved oocytes in the SGA group (16.70±1.78 *vs.* 12.92±0.42, *p*=0.042). The endometrial thickness was significantly lower in the SGA group (10.27±0.23 *vs.* AGA: 11.68±0.17, *p*=0.029). No other differences were observed between AGA and SGA groups.

**Table 3:** COS, laboratorial and clinical outcomes of Small for gestational age (SGA) or Appropriate for gestational age (AGA) infants

	SGA		AGA		*p*
	Mean	SD	Mean	SD	
Total FSH dose	2420.76	48.71	2397.93	36.82	0.724
Estradiol level at hCG day	3897.01	550.35	2324.78	101.86	**0.006**
Follicles	17.05	0.58	15.77	0.78	0.216
Retrieved oocytes	16.70	1.78	12.92	0.42	**0.042**
MII oocytes	12.29	1.35	9.56	0.31	0.051
Fertilization rate	83.68	1.63	84.19	1.20	0.810
Obtained Embryos	8.09	0.31	7.59	0.43	0.385
High-quality embryo rate at day two	81.99	2.11	81.23	1.52	0.783
High-quality embryo rate at day three	62.60	2.46	65.37	1.77	0.387
Blastocyst rate	46.90	2.88	48.47	2.14	0.679
Endometrial thickness	10.27	0.23	11.68	0.17	**0.029**
Implantation rate	71.47	1.16	71.72	0.85	0.781

FSH= Follicle-stimulating hormone, SD= standard deviation. Data was adjusted for maternal age and BMI, number of transferred embryos, number of gestational sacs and number of born infants.

## DISCUSSION

Children conceived by ART represent a substantial proportion of the population, nevertheless, there is still increasing evidence that they have a higher risk of perinatal complications, and the mechanisms behind this have not been well elucidated (Pandey *et al*., 2012; Pinborg *et al*., 2013). The present study showed that specific COS and clinical outcomes affect GW, BW and BL.

The total amount of FSH administered and the estradiol level at hCG day had no influence when perinatal outcomes were singly observed, which corroborated previous observations (Griesinger *et al*., 2008; Sunkara *et al*., 2015). However, when data was classified according to intrauterine growth curve parameters, it was observed that SGA babies came from cycles with significantly higher estradiol level, indicating that response to COS influences intrauterine growth. Elevated estradiol levels have been correlated to impaired embryo implantation potential (Valbuena *et al*., 2001), decrease in the duration of the endometrial receptivity window (Ma *et al*., 2003) and lower pregnancy rates (Mitwally *et al*., 2006). More recently, estradiol levels were also correlated to poorer obstetric and perinatal outcomes (Pereira *et al*., 2017; Royster *et al*., 2016; Sokalska *et al*., 2017). In fact, it has been established that estradiol level >3450 pg/ml on the day of hCG administration is associated with greater odds of preeclampsia and delivery of an SGA infant (Imudia *et al*., 2012). The latter is in accordance to what has been reported by this study.

Our data also showed that BL was negatively influenced by the response to ovarian stimulation, such as numbers of follicles and retrieved oocytes. The relation between COS and BL has been observed mainly in multiple pregnancies studies (Helmerhorst *et al*., 2004; Olivennes *et al*., 1996). Moreover, we also observed a higher number of retrieved oocytes in the SGA group, which indicates that higher response to COS can be prejudicial to proper embryo development, even when results were adjusted for maternal and cycles characteristics. This observation corroborates reports that associated higher number of oocytes retrieved with increased incidence of low birth weight (Baker *et al*., 2015; Kalra *et al*., 2011; Sunkara *et al*., 2015).

Our evidence demonstrated that endometrial thickness could influence not only embryo implantation but also perinatal outcomes. On average, a 1-mm increase in endometrial thickness could prolong pregnancy by 1.4 days and increase baby weight by 28 g. The endometrial thickness was also significantly lower in SGA babies, which supports the importance of this measure before embryo transfer. For the best of our knowledge, correlations between perinatal outcomes and endometrial thickness at hCG day are scarce in the literature and the results obtained emphasizes the need for a more physiologically implantation environment in ART to facilitate the birth of healthier babies.

The strength of this study is that the analyses were multiple adjusted to withdraw multiple pregnancies vies, considering the number of transferred embryos, the number of gestational sacs and the number of born infants as confounder variables. Even though the number of babies analyzed was limited and maternal medical history and social habits were lacking, this is the first Brazilian report of the relation between perinatal outcomes and ICSI characteristics, and it should encourage others to report their own outcomes.

In conclusion, our findings suggest that higher ovarian response to stimulation and suboptimal endometrial development are associated with adverse perinatal outcomes in ICSI cycles.
